# The contribution of white matter changes to clinical phenotype in progressive supranuclear palsy

**DOI:** 10.1007/s00415-024-12662-0

**Published:** 2024-09-02

**Authors:** Maria Francesca Tepedino, Francesco Diana, Filomena Abate, Anna Rosa Avallone, Miriam Caterino, Roberto Erro, Maria Teresa Pellecchia, Renzo Manara, Paolo Barone, Marina Picillo

**Affiliations:** 1 Center for Neurodegenerative Diseases (CEMAND), Department of Medicine, Surgery and Dentistry “Scuola Medica Salernitana”, University of Salerno, Via Allende, 84131 Baronissi, Salerno Italy; 2 Department of Neuroradiology, Azienda Ospedaliera Universitaria San Giovanni di Dio e Ruggi d’Aragona, Salerno, Italy; 3 Interventional Neurology Department, Vall d’Hebron University Hospital, Barcelona, Spain; 4 Neuroradiology Unit, Department of Neurosciences, University of Padua, 35128 Padua, Italy

**Keywords:** Progressive supranuclear palsy, Phenotype, Vascular, MRI

## Abstract

White matter hyperintensities (WMH) are considered magnetic brain imaging (MRI) biomarkers of cerebral small vessel disease but their clinical role in neurodegenerative-related disorders is poorly understood. This study describes the distribution of WMH on brain MRI in Progressive Supranuclear Palsy (PSP) in comparison with Parkinson’s disease (PD) and explores their possible impact on disease’s features. Sixty PSP and 33 PD patients were included. Motor symptoms, cardiovascular risk factors and the age-related white matter changes (ARWMC) score was computed to rate WMH for both groups. Pearson’s correlation and linear or logistic regression analysis were used to check for relationships between ARWMC and PSP clinical scores. The mean (standard deviation) ARWMC total score in the PSP cohort was 4.66 (3.25). Any degree of WMH was present in 68% of PSP (ARWMC +). Compared to ARWMC-, ARWMC + did not have greater disease severity or more cardiovascular risk factors. WMH were frequently localized in fronto-parietal lobes and were mild in severity. Linear regression analysis showed that ARWMC total score was related to the PSP-rating scale, irrespective of age, disease duration and the Charlson modified comorbidity index. Logistic regression analysis confirmed that ARWMC total score was related to the use of wheelchair, irrespective of above-mentioned covariates. Vascular risk factors as well as severity and distribution of WMH did not have an impact on the PSP phenotype. No differences were found with PD patients. Our results suggest that WMH in PSP might be markers of neurodegenerative-related pathology rather than being simple expression of atherosclerotic cerebrovascular changes.

## Introduction

Progressive Supranuclear Palsy (PSP) is a rare, rapidly progressive neurodegenerative disease characterized by accumulation of the four-repeat (4R) isoform of the microtubule-associated protein tau in neurons and glial cells [1]. Clinical characteristics of PSP include early onset of postural instability with falls, vertical gaze palsy, akinesia and cognitive dysfunction. By combining the differential involvement of the four clinical core domains, different phenotypes of the disease can be identified other than the classic PSP–Richardson Syndrome (PSP–RS) [2].

Emerging evidence has suggested that cerebral small vessel disease (CSVD) is associated with greater motor, cognitive and behavioral burden in a variety of neurodegenerative diseases [3, 4]. Autopsy studies have noted a strong relationship between Alzheimer disease’s (AD) pathology and CSVD [5, 6]. Despite severe leukoencephalopathy represents a mandatory exclusion criterion for the diagnosis of idiopathic PSP [2], a recent anatomopathological study reports CSVD was the second most frequent co-pathology found in a cohort of 101 PSP (65%) [7]. White matter hyperintensity (WMH) on magnetic resonance imaging (MRI) T2-weighted sequences is one of the neuroimaging markers of CSVD and it can be quantified with a validated scale, the age-related white matter changes (ARWMC) scale [8, 9]. Indeed, given the coexistence of a variety of comorbidities, different degrees of white matter hyperintensity (WMH) on magnetic resonance imaging (MRI) T2-weighted sequences can be detected in patients with idiopathic PSP. The way such vascular load can contribute to the clinical picture of the disease is unknown since a detailed quantification of clinical burden and phenotype was lacking in this study [7].

As a matter of fact, there is a lack of studies focusing on the association between WMH, the presence of vascular risk factors and disease features in PSP.

Herein, we aimed to describe the burden of WMH in a large, prospective cohort of PSP patients compared to Parkinson’s disease (PD) considering vascular risk factors. Further goal was to analyze the influence of WMH on the clinical presentation of the disease in PSP.

## Methods

### Patients and clinical evaluation

The present study included 67 consecutive subjects with PSP diagnosed according to the International Parkinson and Movement Disorder Society (MDS) criteria and referred to the Center of Neurodegenerative Disease (CEMAND) of the University of Salerno, Italy, from May 2016 to July 2022 [2]. This is part of an ongoing research project aimed at describing clinical and radiological features of PSP [10–12]. Specific exclusion criteria for the present study were the presence of contraindications for MRI or unacceptable quality of the acquired MRI scan or history of chronic migraine. The project was approved by local Ethics Committee and all patients gave written informed consent. Demographic features (sex, age at visit) and disease characteristics (disease duration, MDS core clinical features for the computation of the phenotype) were collected [13, 14]. According to a pre-specified algorithm, patients were divided in PSP-RS and the other variant syndromes of PSP (vPSP) [15]. Severity of the disease was evaluated with the PSP rating scale (PSP-rs) and the MDS-UPDRS-III [16, 17]. Furthermore, a greater severity of disease was assumed if any of the following milestones was present: (1) severe dysphagia (PSP-rs item 13 ≥ 3); (2) unintelligible speech (PSP-rs item 12 ≥ 3); (3) being wheelchair-bound; and (4) dementia based on clinical judgment. Functional autonomy was assessed with the Schwab and England (S&E). Cognitive abilities and behavioral disturbances were screened with the Montreal Cognitive Assessment (MOCA) and Neuropsychiatric inventory (NPI), respectively [18, 19].

Medical history was recorded to define the presence or absence of individual vascular risk factors i.e. hypertension, diabetes mellitus type 2, dyslipidemia, obesity and smoking. Furthermore, the Charlson modified comorbidity index was calculated based on the classical Charlson comorbidity index with the addition of information on the presence of hypertension, hyperlipidemia, smoking and obesity since, unlike diabetes mellitus, they are the vascular risk factors not contained in the original score [20].

A cohort of patients with idiopathic PD, diagnosed according to the MDS criteria, matched by gender, age, age at onset, disease duration and Levodopa Equivalent Daily Dose (LEDD), was retrospectively recruited (PSP:PD = 2:1) [21].

### Brain MRI protocol and vascular scoring

All patients underwent a brain MR with 3 Tesla MAGNETOM Skyra (Siemens Healthcare, Erlangen, Germany), with an imaging protocol including axial Fast Spin-Echo T2-weighted and FLAIR sequences.

WMH were defined as ill-defined hyperintensities larger than 5 mm on both T2 and FLAIR images. The differentiation of lacunes and perivascular spaces was based on size and signal intensity. WMH were rated by an experienced neuroradiologist (FD), blinded to the results of clinical evaluation using the validated visual ARWMC scale. This scale assesses the degree of WMH on a 4-point scale (from the absence of any lesion—score 0, to the presence of confluent lesions—score 3), in five different regions of each hemisphere (frontal, parieto-occipital, temporal, infratentorial and basal ganglia) and ranges from a total score of 0–30 [9].

### Statistical analysis

After checking for normality distribution with the Kolmogorov–Smirnov test, parametric or non-parametric tests were applied to detect differences between groups for continuous variables as appropriate. Complementary, differences in categorical variables were analyzed with *χ*^2^ test.

Pearson’s correlation was performed to explore the relationship between ARWMC total score and PSP-rs, MDS-UPDRS-III, S&E, MOCA and NPI scores. Significance level was set at *p* = 0.01 after correction for multiple comparisons with Bonferroni test. To further verify the relationship between WMH and the clinical burden of the disease, multiple linear regression models were built using any clinical variable showing a significant correlation as dependent variables and the ARWMC total score as independent variable with age, disease duration and the Charlson modified comorbidity index as covariates.

Furthermore, Pearson’s correlation was performed to explore the relationship between ARWMC total score and clinical milestones. Significance level was set at *p* = 0.0125 after correction for multiple comparisons with Bonferroni test. To further verify the relationship between WMH and the clinical milestones, multiple logistic regression models were built using any milestone showing a significant correlation as dependent variables and the ARWMC total score as independent variable with age, disease duration and the Charlson modified comorbidity index as covariates.

Finally, to explore the impact of ARWMC total score on the clinical phenotype, a logistic regression model was built with categorization in PSP–RS or vPSP as dependent variable adjusting for age, disease duration and the Charlson modified comorbidity index as covariates.

The statistical analysis was performed using the Statistical Package for Social Science (SPSS, version 26). All statistical tests were two-tailed, and a *p* ≤ 0.05 was deemed as statistically significant.

## Results

### Demographic and clinical characteristics of the whole PSP cohort and group comparison between patients with or without white matter hyperintensities

**Fig. 1:**
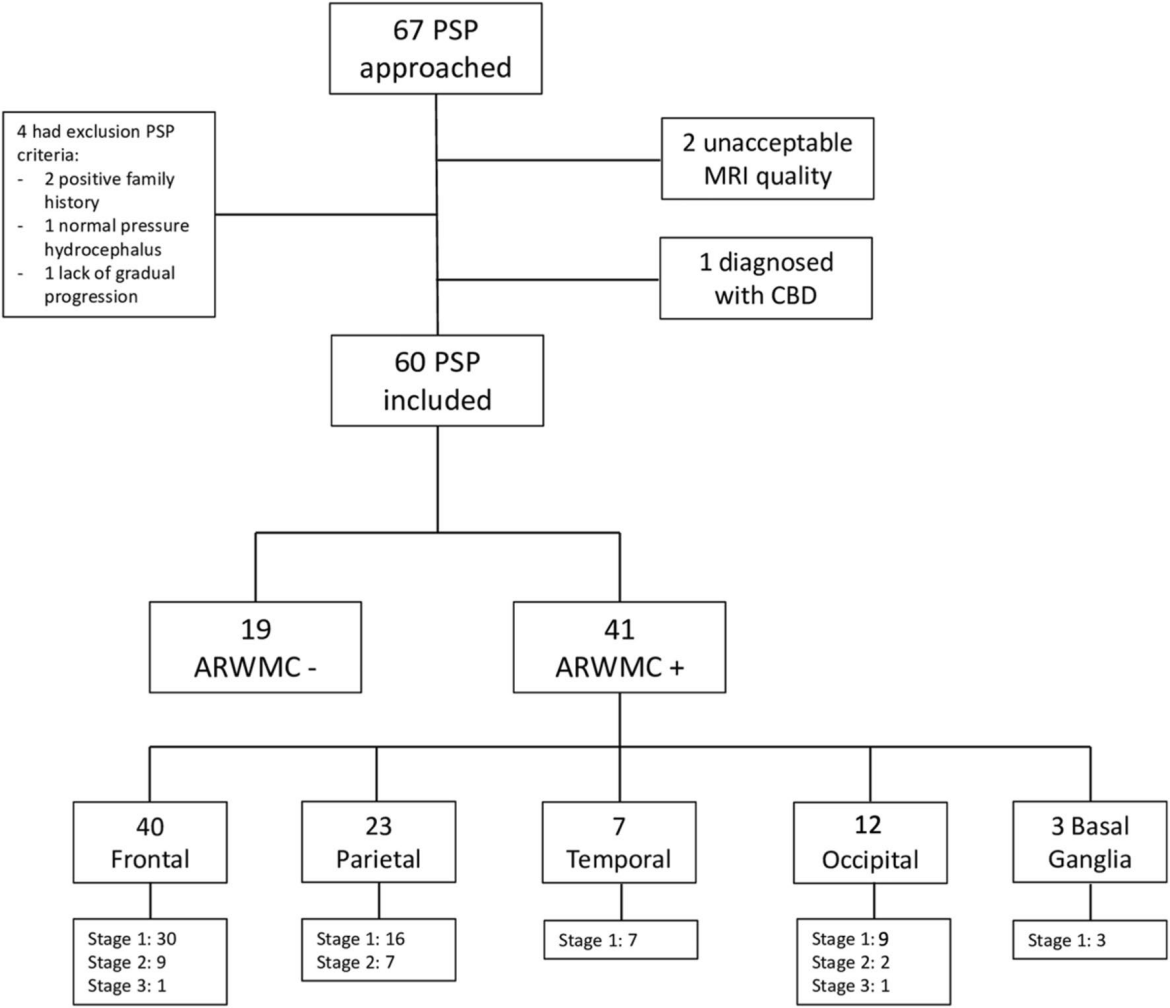
Enrollment flowchart. Legend: *ARWMC* age-related white matter changes; *CBD* corticobasal disease; *MRI* magnetic resonance imaging; *PSP* Progressive Supranuclear Palsy

Sixty out of 67 patients were included in the analysis. Enrollment flowchart is depicted in Fig. 1. All included patients qualified for a diagnosis of probability except for one patient with prevalent oculomotor involvement which—by definition—only qualifies for a diagnosis of possibility.

Demographic, clinical characteristics and vascular risk factors of the PSP sample are shown in Table 1. As expected, the most prevalent phenotype was PSP–RS (70%) with vPSP accounting for 30% of cases. Most subjects were in the early stage of disease as only 11.6% were unable to walk.

**Table 1:** Demographic and clinical characteristics of the enrolled cohort and comparison between PSP with (ARWMC +) and without (ARWMC -) white matter hyperintensities

Characteristics	Total cohort (*n* = 60)	ARWMC = 0 (*n* = 19)	ARWMC > 0 (*n* = 41)	*p*
Demographics				
Age, years	68.5 ± 11.73	69.05 ± 6.7	69.66 ± 5.4	0.711
Age at onset, years	65.5 ± 10.87	66.21 ± 6.8	65.59 ± 6.1	0.726
Disease duration, years	3.68 ± 2.54	2.84 ± 1.6	4.07 ± 2.8	**0.036**
Sex (W), *n* (%)	30 (50)	10 (52.63)	20 (48.7)	0.781
Clinical features				
PSP-rs total score	42.75 ± 20.18	41.50 ± 13.7	42.15 ± 15.67	0.880
MDS-UPDRS-III total score	43.75 ± 20.91	43.11 ± 18.1	41.29 ± 15.4	0.706
S&E	52.5 ± 22.96	19.28 ± 4.42	24.65 ± 3.85	0.654
MOCA total score	18.46 ± 7.58	17.91 ± 5.1	17.60 ± 4.8	0.829
NPI total score	10.50 ± 9.67	9.36 ± 9.5	12.06 ± 10	0.481
MDS-PSP phenotypes, *n* (%)	PSP-RS 42 (70) vPSP 18 (30) PSP-P 7 (38.8) PSP-PGF 7 (38.8) PSP-F 3 (16.6) PSP-OM 1 (5.5)	PSP-RS 15 (79) vPSP 4 (21) PSP-F 2 (50) PSP-P 1 (25) PSP-PGF 1 (25)	PSP-RS 27 (65.9) vPSP 14 (34.1) PSP-P 6 (42.8) PSP-PGF 6 (42.8) PSP-OM 1 (7.1) PSP-F 1 (7.1)	0.303
Milestones				
Severe dysphagia, *n* (%)	3 (5)	1 (5.26)	2 (4.87)	0.946
Unintelligible speech, *n* (%)	11 (18.3)	3 (15.78)	8 (19.51)	0.732
Wheelchair-bound, *n* (%)	7 (11.6)	2 (10.5)	5 (12.5)	0.999
Dementia, *n* (%)	16 (26.7)	5 (26.31)	11 (26.82)	0.973
Vascular risk factors				
Charlson modified comorbidity index	4.07 ± 1.32	3.63 ± 1.3	4.27 ± 1.3	0.084
Diabetes type 2, *n* (%)	10 (16.66)	3 (15.78)	7 (17.07)	0.805
Hypertension, *n* (%)	31 (51.6)	8 (42.1)	23 (56)	0.188
Hyperlipidemia, *n* (%)	14 (23.3)	2 (10.5)	12 (29.2)	0.080
Smoking, *n* (%)	12 (20)	2 (10.5)	10 (24.3)	0.129
Obesity, *n* (%)	4 (6.6)	2 (10.5)	2 (4.8)	0.414

Data are shown in mean ± standard deviation unless otherwise specified

*ARWMC* age related white matter change; *CI* comorbidity index; *LEDD* levodopa equivalent dose; *MDS-UPDRS-III* Movement Disorder Society-Unified Parkinson’s Disease Rating Scale part III; *MOCA* Montreal Cognitive Assessment; *n* number; *NPI* Neuropsychiatric inventory; *PSP* Progressive Supranuclear Palsy; *PSP-rs* PSP rating scale; *S&E* Schwab and England; W: women

Significant results are highlighted in bold (p ≤ 0.05)

Nineteen patients (31.7%) did not present any WMH (ARWMC -), while 41 of them (68.3%) presented any degree of WMH (ARWMC +). As for ARWMC +  27 (65.9%) were PSP–RS and 14 (34.1%) were vPSP. The two groups presented similar features except for a significant longer disease duration (*p* = 0.036) and a tendency towards higher hyperlipidemia rates (*p* = 0.080) as well as greater comorbidity index (*p* = 0.084) in ARWMC + (Table 1).

### Comparisons of demographic features, clinical characteristics and WMH between PSP ad PD cohort

Table 2 provides details on the comparison between PSP and PD cohort. No significant differences were shown between the groups according to gender, age, age at onset, disease duration and LEDD. As expected, PSP presented a greater disease severity measured with MDS-UPDRS-III total score compared to PD (*p* < 0.001). No significant differences in terms of prevalence of classic vascular risk factors were found between groups, excepted for the Charlson modified comorbidity index which was higher in PSP (*p* = 0.026).

**Table 2:** Comparison of demographic, clinical and radiological characteristics between PSP and PD subjects

Characteristic	PSP (*n* = 60)	PD (*n* = 33)	*p*
Demographics and clinical features			
Age	69.47 ± 5.81	67.81 ± 8.12	0.262
Age at onset	65.78 ± 6.34	63.28 ± 7.85	0.101
Disease duration	3.68 ± 2.54	4.59 ± 2.75	0.116
Sex (W), *n* (%)	30 (50)	10 (30.3)	0.066
MDS-UPDRS-III total score, median (IQR)	41.5 (22)	19.5 (6)	< 0.001*
LEDD, median (IQR)	350 (408)	427.5 (359)	0.269
Vascular risk factors			
Charlson modified comorbidity index, median (IQR)	4 (2)	3 (2)	0.026*
Diabetes type 2, *n* (%)	10 (16.66)	2 (7.69)	0.237^§^
Hypertension, *n* (%)	31 (51.66)	12 (46.15)	0.486^§^
Hyperlipidemia, *n* (%)	14 (23.33)	4 (15.38)	0.347^§^
Smoking, *n* (%)	12 (20)	4 (15.38)	0.194^§^
Obesity, *n* (%)	4 (6.66)	0	0.178^§^
Radiological features			
ARWMC + , *n* (%)	41 (68.3%)	20 (60.6%)	0.453
ARWMC total score, median (IQR)	2 (4)	2 (3)	0.131
ARWMC frontal, *n* (%)	40 (66.7)	19 (57.6)	0.384
ARWMC parietal, *n* (%)	23 (38.3)	8 (24.2)	0.168
ARWMC temporal, *n* (%)	7 (11.7)	4 (12.1)	0.948
ARWMC occipital, *n* (%)	12 (20)	2 (6.1)	0.072
ARWMC basal ganglia, *n* (%)	3 (5)	3 (9.1)	0.442

Data are shown in mean ± standard deviation unless otherwise specified

*ARWMC* age related white matter change; *LEDD* levodopa equivalent dose; MDS-UPDRS-III: Movement Disorder Society-Unified Parkinson’s Disease Rating Scale part III; *PSP* Progressive Supranuclear Palsy; W: women

^§^Data are available for 26 PD patients

Significant results are highlighted in bold (p ≤ 0.05)

As within the PSP cohort, 60% of PD presented any degree of ARWMC (ARWMC +). The mean ARWMC total score (± standard deviation) was 2.33 (± 3.9), with no significant differences observed compared to PSP patients also in terms of spatial localization.

### White matter hyperintensity features and correlation with severity of disease in PSP

**Fig. 2:**
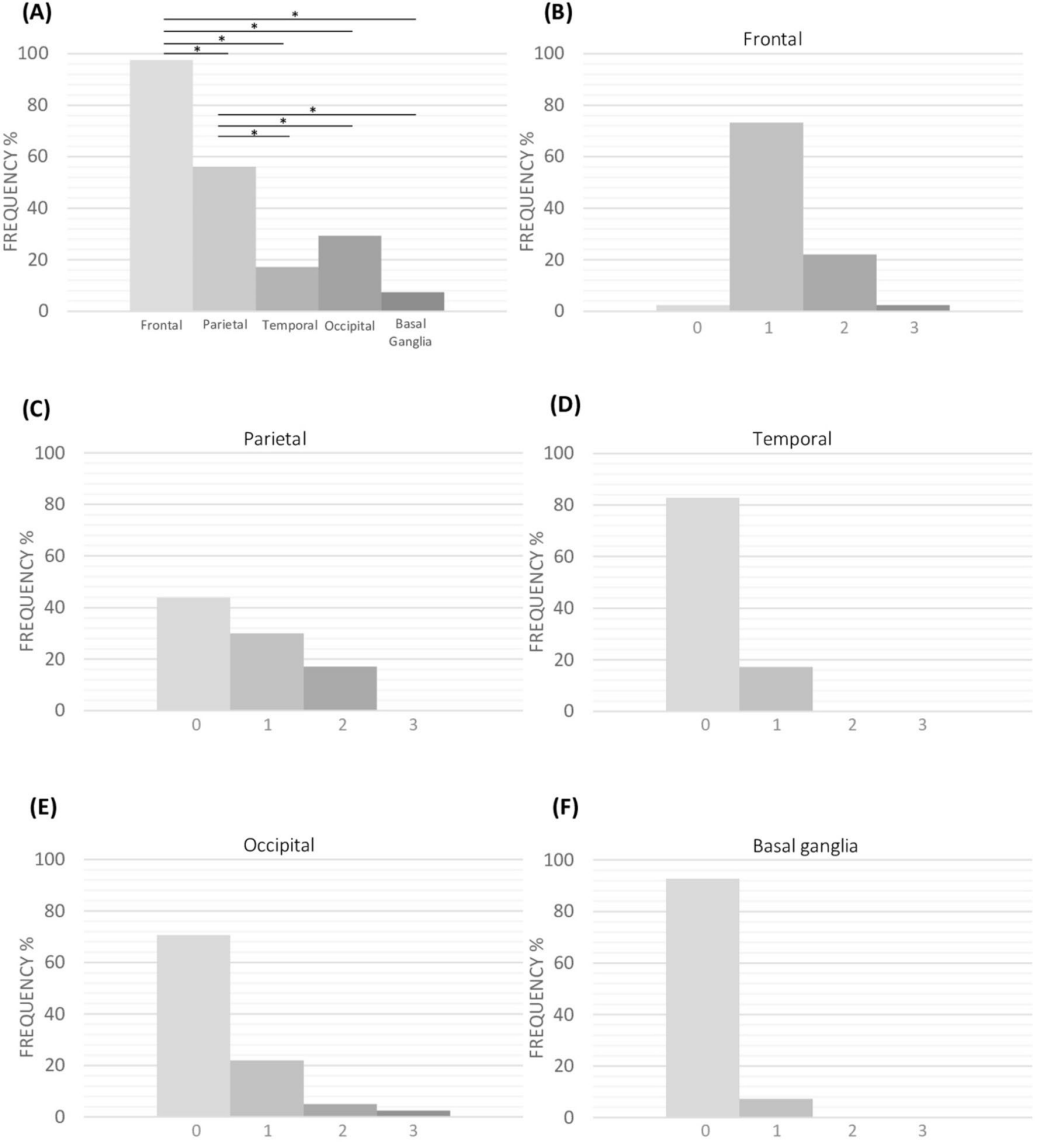
Spatial distribution of white matter hyperintensities on brain MRI

Among patients ARWMC + , the mean ARWMC total score was 4.66 (± 3.25). WMH were predominantly distributed in frontal and parietal lobes (97.6% and 56.1%, respectively) and were mild-to-moderate in severity (scoring 1 or 2 in 97.5% in frontal, 100% in parietal and temporal, 91.6% in occipital and 100% in basal ganglia) (Fig. 2A–F).

Pearson’s correlation showed a significant positive correlation between ARWMC total score and PSP-rs total (*r* = 0.501, *p* = 0.001) and MDS-UPDRS-III (*r* = 0.448, *p* = 0.008). No significant correlations were found between ARWMC total score and S&E, MOCA or NPI total score.

**Fig. 3:**
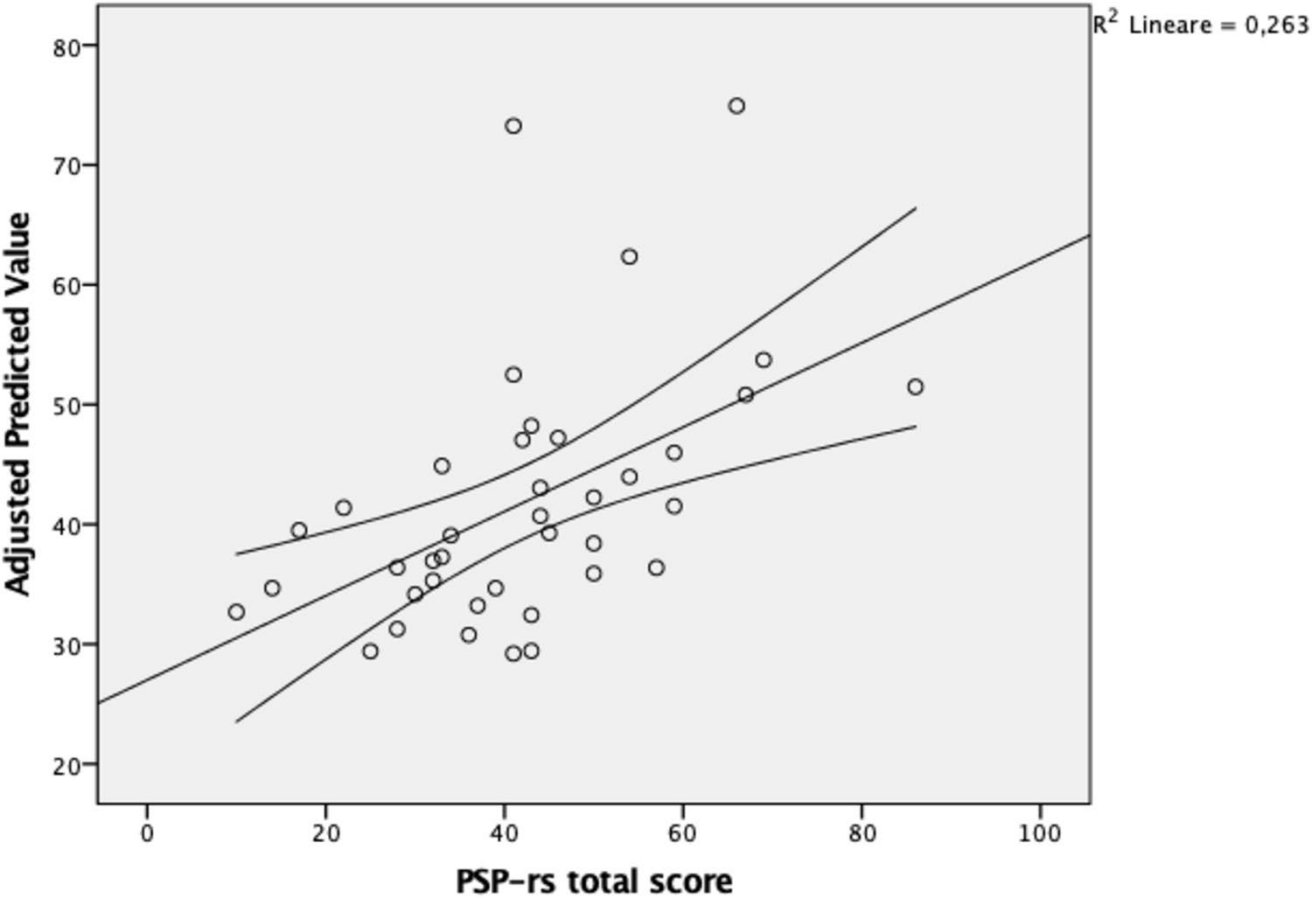
Linear regression analysis between ARWMC total score and PSP-rs. Legend: *PSP-rs* Progressive Supranuclear Palsy- rating scale

Linear regression analysis confirmed that ARWMC total score was related to the PSP-rs (*β* = 0.312, 95% CI 0.161–3.175, *p* = 0.031), irrespective of age, disease duration and the Charlson modified comorbidity index (Fig. 3). Complementary, a trend towards significance was demonstrated for the relationship between the ARWMC total score and the MDS-UPDRS-III (*β* = 0.289, 95% CI − 0.074–3.131, *p* = 0.061) considering age, disease duration and the Charlson modified comorbidity index as covariates.

Pearson’s correlation showed a significant direct relationship between the ARWMC total score and unintelligible speech (*r* = 0.397, *p* = 0.0120) as well as the use of wheelchair (*r* = 0.550, *p* < 0.001). No significant correlations were found between the ARWMC total score and the other milestones of disease. Logistic regression analysis confirmed that ARWMC total score was related to the use of wheelchair (OR = 1.305, 95% CI 1.008–1.689, *p* = 0.043), irrespective of age, disease duration and the Charlson modified comorbidity index. On the other hand, no relationship was confirmed for unintelligible speech.

### Relationship between WMH and PSP phenotypes

Within the ARWMC + , 27 (65.8%) qualified for PSP-RS and 14 (34.1%) for vPSP. No differences were detected in demographic variables between groups. PSP–RS presented greater PSP-rs total score (*p* = 0.001) and lower S&E (*p* = 0.008) compared to vPSP. The two groups disclosed a similar distribution of vascular risk factors as well as severity and distribution of WMH (Table 3). Logistic regression model showed no relationship between the ARWMC total score and clinical phenotype covarying for age, disease duration and comorbidity index.

**Table 3:** Comparison of demographic, clinical and radiological characteristics between PSP-RS and vPSP within PSP with white matter hyperintensities (ARWMC +)

	PSP-RS (*n* = 27)	vPSP (*n* = 14)	*p*
Demographics			
Age, years	69.52 ± 6.13	69.93 ± 3.81	0.821
Age at onset, years	65.26 ± 6.89	66.21 ± 4.56	0.644
Disease duration, years	4.26 ± 2.87	3.71 ± 2.75	0.563
Sex (W), *n* (%)	14 (51.8)	6 (42.85)	0.744
Clinical features			
PSP-rs total score	47.58 ± 13.95	31.31 ± 13.47	**0.001**
MDS-UPDRS-III total score	44 ± 16.45	35.64 ± 11.79	0.142
S&E	46.3 ± 24.2	67.14 ± 19.77	**0.008**
MOCA total	17.30 ± 4.7	18.18 ± 5.17	0.615
NPI total score	12.92 ± 10.56	10.33 ± 9.48	0.621
Vascular risk factors			
Charlson modified comorbidity index	4.19 ± 1.36	4.43 ± 1.22	0.578
Diabetes Mellito type 2	3 (11.11)	4 (28.57)	0.157
Hypertension, *n* (%)	14 (51.85)	9 (64.28)	0.501
Hyperlipidemia, *n* (%)	6 (22.22)	6 (42.85)	0.270
Smoking, *n* (%)	5 (18.51)	5 (35.71)	0.687
Obesity, *n* (%)	2 (7.4)	0 (0)	0.539
Radiologic features			
ARWMC total score	4.15 ± 2.94	5.64 ± 3.69	0.166
ARWMC frontal, *n* (%)	26 (96.29)	14 (100)	1
ARWMC parietal, *n* (%)	13 (48.14)	10 (71.42)	0.196
ARWMC temporal, *n* (%)	4 (14.81)	3 (21.42)	0.673
ARWMC occipital, *n* (%)	7 (25.92)	5 (35.71)	0.719
ARWMC basal ganglia, *n* (%)	1 (3.7)	2 (14.28)	0.265

Data are shown in mean ± standard deviation unless otherwise specified

*ARWMC* age related white matter change; *LEDD* levodopa equivalent dose; *MDS-UPDRS-III* Movement Disorder Society-Unified Parkinson’s Disease Rating Scale part III; *MOCA* Montreal Cognitive Assessment; *n* number; *NPI* Neuropsychiatric inventory; *PSP-RS* Progressive Supranuclear Palsy Richardson’s Syndrome; *PSP-rs* PSP rating scale; *vPSP* the other variant syndromes of PSP; *S&E* Schwab and England; W:women

Significant results are highlighted in bold (p ≤ 0.05)

## Discussion

Herein, we demonstrated that WMH detected with brain MRI are highly prevalent and represent a significant determinant of the clinical burden in PSP, also considering the role of age, disease duration and common vascular risk factors. We also showed the severity and location of WMH in PSP are similar to a comparable cohort of PD.

In our series of patients, any degree of WMH was as prevalent as nearly 70% in clinically diagnosed PSP. ARWMC + presented similar demographic and clinical features as well as similar distribution of comorbidities and vascular risk factors as compared to ARWMC- except for longer disease duration and a tendency towards higher hyperlipidemia rates in ARWMC + . The overall severity of white matter changes was low (mean score: 4.66, range: 0–30). Distribution of WMH was predominant in fronto-parietal lobes with each region showing mild-to-moderate staging in most cases. Such data are in line with recent findings from a neuropathological study where CSVD was the second most common co-pathology (65%) in PSP and mostly mild-to-moderate in severity (stage I and II) [7]. In the same study the authors failed to demonstrate an effect of CSVD co-pathology on PSP clinical features. However, despite including a similar number of patients (101 versus 60), the study lacked of specific rating scales to quantify the disease burden and clinical data were extracted from patients’ charts in the form of disease milestones. Furthermore, common vascular risk factors and comorbidities were not detailed [7]. In our study at the time of the brain MRI each patient underwent an extensive clinical evaluation including the PSP-rs as measure of global severity of the disease, the MDS-UPDRS-III, scoring parkinsonism and more in general akinesia, the S&E evaluating disability associated with the disease and the MOCA and the NPI, evaluating the cognitive and behavioral burden of disease, respectively. Correlation analysis showed a significant positive relationship between the total white matter changes and the global clinical burden as assessed with the PSP-rs total as well as with parkinsonism as rated with the MDS-UPDRS-III. Then linear regression analysis confirmed such relationship was significant for the PSP-rs, irrespective of age, disease duration and vascular comorbidities. Further corroborating the notion that global white matter changes may shape the clinical picture in PSP, we also disclosed a significant relationship between WMH and the use of wheelchair. In detail, each point increase in the ARWMC score determined a 1.3-folded risk of being wheelchair-bound irrespective of age, disease duration and comorbidity index.

Finally, we attempted to understand whether white matter changes had a role in depicting PSP phenotype. However, we failed to demonstrate a relationship between the degree of WMH and the attribution to either RS or vPSP group covarying for age, disease duration and comorbidities. Overall, our results suggest that white matter changes may contribute to the global severity of the disease rather than to the specific phenotype, which remains related to selective regional neurodegenerative processes.

In line with previous findings in PD [22], our data suggests that white matter changes might contribute to PSP global burden by altering the integrity and therefore increasing the vulnerability of cortico-striatal and cortico-cortical connections, especially at frontal and parietal level. Nonetheless, the etiology of WMH in PSP remains unclear [7]. In fact, areas with altered signal intensity in the white matter are frequently encountered in brain imaging studies in the elderly population and are generally associated with cardiovascular risk factors [23]. Accordingly, different clinical studies have associated a variety of comorbidities and vascular risk factors to PSP without describing potentially related white matter changes at brain MRI [24–26]. Intriguingly, in the present study the number of comorbidities as well as specific cardiovascular risk factors were not significantly increased in patients with white matter changes. Thus, the presence of other processes determining white matter changes cannot be completely ruled out, such as Wallerian degeneration due to concomitant grey matter atrophy, brain–blood barrier dysfunction, altered cell metabolic pathways, glial injury or inflammation [27, 28]. Indeed, white matter changes in PD have previously been found in areas with prevalent cortical atrophy, associating with subsequent motor and non-motor outcomes [29, 30]. As a matter of fact and similar to PSP, 60% of our PD cohort showed any degree of WMH without presenting greater prevalence of classic vascular risk factors. In line with previous findings [27, 31, 32], our data suggests that both for PSP and PD, WMH may represent alternative neurodegeneration-related changes beyond CSVD. We acknowledge PSP presented greater Charlson modified comorbidity index and speculate it may be due to other non-cardiovascular comorbidities.

Giving the lack of associations with vascular risk factors, we speculate white matter changes might represent a common, intrinsic feature of PSP instead of being a simple secondary event associated with comorbidities. The relationship between CSVD and Frontotemporal lobar degeneration (FTLD)-tau has been investigated. Recent anatomopathological evidence showed a co-localization of CSVD with white matter tau-pathology predominant in frontal and temporal lobes [33]. Such findings open a new, more complex scenario. Vascular and vessel pathology may indeed have an influence on the clinical manifestation of non-AD tauopathies [34]. Alternatively, or complementary, white matter degeneration and demyelination may be triggered by severe involvement [34, 35]. The co-localization of both WMH and tau-pathology in frontal lobes in PSP may support either or both such hypothesis. Deep anatomopathological and in vivo imaging definition of the relationship between white matter disease and tau-pathology is warranted as white matter disease may represent a complementary target to tauopathy for potential disease-modifying treatments.

We acknowledge our study lacks neuropathological confirmation of both PSP diagnosis and white matter disease. However, clinical diagnosis was achieved through a rigorous application of the MDS criteria, all patients have been followed after the inclusion in the study and the diagnosis was confirmed after at least one year follow-up. Also, imaging assessment did not include volumetric measures or other sequences to evaluate other markers of white matter changes possibly contributing to ageing and neurodegeneration, such as cerebral microbleeds [36]. As for the description of the white matter changes we used the ARWMC scoring, one of the most reliable imaging marker of vascular load, which was computed by an experienced radiologist blinded to patients’ clinical evaluation and phenotype. Despite lacking neuropathological confirmation, our study includes an extensive clinical description of the disease allowing a detailed correlation analysis. The lack of longitudinal follow-up is a limitation of the study but can be a starting point for future research.

In conclusion, in the present single-center prospective study we showed white matter changes are present in nearly two third of PSP patients irrespective of comorbidities and vascular risk factors and are more frequently located in fronto-parietal lobes being mild-to-moderate in severity. By applying an extensive clinical description of our cohort, we also demonstrated white matter changes are significantly related with the global burden of neurological impairment as well as with clinical milestones but not with the disease phenotype (RS versus vPSP). Deep knowledge of the mutual relationship between tau pathology and white matter disease is warranted as the latter can be considered a potential complementary target for disease-modifying treatments.

## Author contributors

M.P., M.F.T. and F.D. wrote the main manuscript. M.P. and M.F.T. performed the statistical analysis. M.P., M.F.T., F.D., F.A., A.R.A., M.C., R.E., M.T.P., R.M., P.B. checked the data quality and reviewed the manuscript. M.P., M.F.T., F.D., F.A., A.R.A., M.C., R.E., M.T.P., R.M., P.B. collected the data. All the authors have read and approved the final manuscript.

## Data availability statement

The dataset of the present study is available from the corresponding author upon reasonable request.

## References

[1] Dickson DW (1999) Neuropathologic differentiation of progressive supranuclear palsy and corticobasal degeneration. J Neurol 246(Suppl 2):II6-15 10525997 10.1007/BF03161076

[2] Hoglinger GH, Respondek G, Stamelou M et al (2017) Clinical diagnosis of progressive supranuclear palsy: the movement disorder society criteria. Mov Disord 32(6):853–64 28467028 10.1002/mds.26987 PMC5516529

[3] Malek N, Lawton MA, Phil M et al (2015) Vascular disease and vascular risk factors in relation to motor features and cognition in early Parkinson’s disease. Mov Dis 31(10):1518–1526 10.1002/mds.26698 PMC5082556 27324570

[4] Shibata K, Sugiura M, Nishimura Y, Sakura H (2019) The effect of small vessel disease on motor and cognitive function in Parkinson’s disease. Clin Neurol and Neurosurg 182:58–62 10.1016/j.clineuro.2019.04.029 31078957

[5] Moghekar A, Kraut M, Elkins W et al (2012) Cerebral white matter disease is associated with Alzheimer pathology in a prospective cohort. Alzheimers Dement 8(50):S71–S77 23021624 10.1016/j.jalz.2012.04.006 PMC3474974

[6] Kim HW, Hong J, Jeon JC (2020) Cerebral small vessel disease and alzheimer’s disease: a review. Front Neurol 11:927 32982937 10.3389/fneur.2020.00927 PMC7477392

[7] Lukic MJ, Kurz C, Respondek G et al (2020) The MDS-endorsed PSP study group. copathology in progressive supranuclear palsy: does it matter? Mov Disord 35(6):974–993 10.1002/mds.28011 32125724

[8] Chen X, Wang J, Shan Y et al (2018) Cerebral small vessel disease: neuroimaging markers and clinical implication. J Neurol 266(10):2347–2362 30291424 10.1007/s00415-018-9077-3

[9] Wahlund LO, Barkhof F, Fazekas F et al (2001) A new rating scale for age-related white matter changes applicable to MRI and CT. Stroke 32(6):1318–1322 11387493 10.1161/01.str.32.6.1318

[10] Picillo M, Cuoco S, Tepedino MF et al (2019) Motor, cognitive and behavioral differences in MDS PSP phenotypes. J Neurol 266(7):1727–1735 30989369 10.1007/s00415-019-09324-x

[11] Picillo M, Erro R, Cuoco S et al (2018) MDS PSP criteria in real-life clinical setting: motor and cognitive characterization of subtypes. Mov Disord 33(8):1361–1365 29984518 10.1002/mds.27408

[12] Picillo M, Tepedino MF, Abate F et al (2020) Midbrain MRI assessments in progressive supranuclear palsy subtypes. J Neurol Neurosurg Psychiatry 91(1):98–103 31527182 10.1136/jnnp-2019-321354

[13] Respondek G, Höglinger GU (2016) The phenotypic spectrum of progressive supranuclear palsy. Parkinsonism Relat Disord 22(Suppl. 1):S34–S36 26421392 10.1016/j.parkreldis.2015.09.041

[14] Tomlinson CL, Stowe R, Patel S et al (2010) Systematic review of levodopa dose equivalency reporting in Parkinson’s disease. Mov Disord 25(15):2649–2653 21069833 10.1002/mds.23429

[15] Grimm M-J, Respondek G, Stamelou M et al (2019) Movement Disorder Society-endorsed PSP Study Group. how to apply the movement disorder Society criteria for diagnosis of progressive supranuclear palsy. Mov Disord 34:1228–1232 30884545 10.1002/mds.27666 PMC6699888

[16] Golbe LI, Ohman-Strickland PA (2007) A clinical rating scale for progressive supranuclear palsy. Brain 130(Pt 6):1552–1565 17405767 10.1093/brain/awm032

[17] Antonini A, Abbruzzese G, Ferini-Strambi L et al (2013) Validation of the Italian version of the movement disorder society-unified Parkinson’s disease rating scale. Neurol Sci 34(5):683–687 22678179 10.1007/s10072-012-1112-z

[18] Cummings JL (1997) The Neuropsychiatric Inventory: assessing psychopathology in dementia patients. Neurol 48(5 Suppl 6):S10–S16 10.1212/wnl.48.5_suppl_6.10s 9153155

[19] Nasreddine ZS, Phillips NA, Bedirian V et al (2005) The montreal cognitive assessment, MoCA: a brief screening tool for mild cognitive impairment. J Am Geriatr Soc 53:695–699 15817019 10.1111/j.1532-5415.2005.53221.x

[20] Diederichs CP, Wellmann J, Bartels DB et al (2012) How to weight chronic disease in multimorbidity indices? Development of a new method on the basis of individual data from live population-based studies. J Clin Epidemiol 65(6):679–685 22424984 10.1016/j.jclinepi.2011.11.006

[21] Postuma RB, Berg D, Stern M et al (2015) MDS clinical diagnostic criteria for Parkinson’s disease. Mov Disord 30:1591–1601 26474316 10.1002/mds.26424

[22] Moccia M, Tedeschi E, Ugga L et al (2016) White matter changes and the development of motor phenotypes in de novo Parkinson’s Disease. J Neurol Sci 367:215–219 27423590 10.1016/j.jns.2016.06.015

[23] Kotagal V, Albin RL, Müller MLTM et al (2014) Modifiable cardiovascular risk factors and axial motor impairments in Parkinson disease. Neurology 82(17):1514–1520 24682965 10.1212/WNL.0000000000000356 PMC4011463

[24] Rabadia SV, Litvan I, Juncos J et al (2019) ENGENE PSP study. hypertension and progressive supranuclear palsy. Parkinsonism Relat Disord 66:166–170 31420308 10.1016/j.parkreldis.2019.07.036

[25] Greten S, Wegner F, Jensen I et al (2024) The comorbidity and co-medication profile of patients with progressive supranuclear palsy. J Neurol 271(2):782–793 37803149 10.1007/s00415-023-12006-4 PMC10827866

[26] Zella MAS, Bartig D, Herrmann L et al (2020) Hospitalization rates and comorbidities in patients with progressive supranuclear palsy in Germany from 2010 to 2017. Clin Med 9(8):2454 10.3390/jcm9082454 PMC7465231 32751888

[27] Wardlaw JM, Valdes Hernandez MC, Munoz-Maniega S (2015) What are white matter hyperintensities made of? Relevance to vascular cognitive impairment. Am Heart J 4:001140 10.1161/JAHA.114.001140 PMC4599520 26104658

[28] Bohnen NI, Albin RL (2011) White matter lesions in Parkinson disease. Nat Rev Neurol 7(4):229–323 21343896 10.1038/nrneurol.2011.21 PMC3739056

[29] Danti N, Toschi SD et al (2015) Cortical thickness in de novo patients with Parkinson disease and mild cognitive impairment with consideration of clinical phenotype and motor laterality. Eur J Neurol 22(12):1564–1572 26212370 10.1111/ene.12785

[30] Noh SW, Han NY, Mun CW et al (2014) Analysis among cognitive profiles and gray matter volume in newly diagnosed Parkinson’s disease with mild cognitive impairment. J Neurol Sci 34(1–2):210–213 10.1016/j.jns.2014.09.049 25451006

[31] Compta Y, Buongiorno M, Bargalló N et al (2016) White matter hyperintensities, cerebrospinal amyloid-beta and dementia in Parkinson’s disease. J Neurol Sci 367:284–290 27423605 10.1016/j.jns.2016.06.009

[32] Joki H, Higashiyama Y, Nakae Y et al (2018) White matter hyperintensities on MRI in dementia with Lewy bodies, Parkinson’s disease with dementia, and Alzheimer’s disease. J Neurol Sci 385:99–104 29406924 10.1016/j.jns.2017.12.018

[33] Thal DR, von Arnim CAF, Griffin WST et al (2015) Frontotemporal lobar degeneration FTLD-tau: preclinical lesions, vascular and Alzheimer-related co-pathologies. J Neural Transm 122(7):1007–1018 25556950 10.1007/s00702-014-1360-6 PMC6655426

[34] Englund E (1998) Neuropathology of white matter changes in Alzheimer’s disease and vascular dementia. Dement Geriatr Cogn Disord 9(Suppl 1):6–12 9716238 10.1159/000051183

[35] Leys D, Pruvo JP, Parent M et al (1991) Could Wallerian degeneration contribute to “leuko-araiosis” in subjects free of any vascular disorder? J Neurol Neurosurg Psychiatry 54:46–50 2010759 10.1136/jnnp.54.1.46 PMC1014298

[36] Wardlaw JM, Smith EE, Biessels GJ et al (2013) Neuroimaging standards for research into small vessel disease and its contribution to ageing and neurodegeneration. Lancet Neurol 12(8):822–838 23867200 10.1016/S1474-4422(13)70124-8 PMC3714437

